# Fire and forage quality: Postfire regrowth quality and pyric herbivory in subtropical grasslands of Nepal

**DOI:** 10.1002/ece3.8794

**Published:** 2022-04-13

**Authors:** Shyam Kumar Thapa, Joost F. de Jong, Anouschka R. Hof, Naresh Subedi, Laxmi Raj Joshi, Herbert H. T. Prins

**Affiliations:** ^1^ Wildlife Ecology and Conservation Group Wageningen University and Research Wageningen The Netherlands; ^2^ 221016 National Trust for Nature Conservation Lalitpur Nepal; ^3^ Department of Wildlife, Fish and Environmental Studies Swedish University of Agricultural Sciences (SLU) Umeå Sweden; ^4^ Animal Sciences Group Wageningen University Wageningen The Netherlands

**Keywords:** burned grassland, Cwa climate, grazer and mixed feeders, grazing lawns, Mesofaunal deer assemblage, nutrients

## Abstract

Fire is rampant throughout subtropical South and Southeast Asian grasslands. However, very little is known about the role of fire and pyric herbivory on the functioning of highly productive subtropical monsoon grasslands lying within the Cwa climatic region. We assessed the temporal effect of fire on postfire regrowth quality and associated pyric‐herbivory in the subtropical monsoon grasslands of Bardia National Park, Nepal. Every year, grasslands are burned as a management intervention in the park, especially between March and May. Within a week after fire, at the end of March 2020, we established 60 m × 60 m plots within patches of burned grassland in the core area of the Park. We collected grass samples from the plots and determined physical and chemical properties of the vegetation at regular 30‐day intervals from April to July 2020, starting from 30 days after fire to assess postfire regrowth forage quality. We counted pellet groups of cervids that are abundant in the area for the same four months from 2 m × 2 m quadrats that were permanently marked with pegs along the diagonal of each 60 m × 60 m plot to estimate intensity of use by deer to the progression of postfire regrowth. We observed strong and significant reductions in crude protein (mean value 9.1 to 4.1 [55% decrease]) and phosphorus (mean value 0.2 to 0.11 [45% decrease]) in forage collected during different time intervals, that is, from 30 days to 120 days after fire. Deer utilized the burned areas extensively for a short period, that is, up to two months after fire when the burned areas contained short grasses with a higher level of crude protein and phosphorus. The level of use of postfire regrowth by chital (*Axis axis*) differed significantly over time since fire, with higher intensity of use at 30 days after fire. The level of use of postfire regrowth by swamp deer (*Rucervus duvaucelii*) did not differ significantly until 90 days after fire, however, decreased significantly after 90 days since fire. Large‐scale single event fires, thus, may not fulfil nutritional requirements of all species in the deer assemblage in these subtropical monsoon grasslands. This is likely because the nutritional requirements of herbivores differ due to differences in body size and physiological needs—maintenance, reproduction, and lactation. We recommend a spatiotemporal manipulation of fire to reinforce grazing feedback and to yield forage of high quality for the longest possible period for a sustainable high number of deer to maintain a viable tiger population within the park.

## Graphical abstract

Given the widespread use of fire as a cost‐effective grassland management tool in subtropical monsoon grasslands in the Cwa climate region, it is important to realize that the positive benefit of a single event fire for the conservation of large herbivores is time specific, as the effect of fire on forage quality perhaps lasts only for short period.

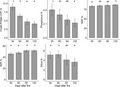

## 1 INTRODUCTION

Fire is an important component of grassland ecosystems and is considered a cost‐effective management tool to prevent the successional change of grassland toward forests (Archibald, 2008; van Langevelde, 2003a, 2003b; Ratnam et al., 2011, 2019). Numerous studies indicate that fire‐grazing interactions, also termed “pyric herbivory,” are complex and can modify grassland systems by creating mosaics of vegetation that vary in structure, composition, quality, and quantity (Allred et al., 2011; Harrison et al., 2003; Klop et al., 2007; Sabiiti et al., 1992; Thapa, Thapa, et al., 2021; Trollope, 2011). With these notable impacts, many wildlife managers consider pyric herbivory essential for the conservation and management of savannas and other grasslands, including the remaining subtropical grasslands of Asia. In Nepal and India, few subtropical monsoon grasslands remain at the foot of the Himalayas. These grasslands rank among the world's most productive (Lehmkuhl, 1994; Peet, Watkinson, Bell, & Kattel, 1999) and represent the globally important ecoregion “Terai‐Duar Savanna and Grasslands” (Olson & Dinerstein, 2002). These grasslands are burned annually by park staff and local people to stimulate new grass growth, enhance grazing opportunities, increase the availability of good thatching grass, remove woody encroachment, increase visibility, and reduce fire hazards (Lehmkuhl, 1994; Peet, Watkinson, Bell, & Kattel, 1999). In addition, much burning takes place due to accidents and lightning. Identifying the effects of fire on forage quality and associated pyric herbivory in the subtropical grasslands is paramount for wildlife conservation and management because the Asian subtropical monsoon grasslands host many threatened and endangered vertebrates, for example, Bengal florican (*Houbaropsis bengalensis*), hispid hare (*Caprolagus hispidus*), wild water buffalo (*Bubalus arnee*), Greater one‐horned rhinoceros (*Rhinoceros unicornis*), Royal Bengal tiger (*Panthera tigris*), hog deer (*Axis porcinus*), and swamp deer (*Rucervus duvaucelii*).

The role of fire, herbivory, and their interaction effect on ecosystem functioning have been extensively studied through experiments and modeling on African savannas and North American prairies (Allred et al., 2011; Archibald & Bond, 2004; Archibald & Hempson, 2016; Donaldson et al., 2018; Fuhlendorf et al., 2009; Klop et al., 2007; Leverkus et al., 2018; Raynor et al., 2016; Van de Vijver et al., 1999; Veach et al., 2014). However, very little is known about the role of fire and pyric herbivory on functioning of the highly productive subtropical monsoon grasslands lying within the Cwa climatic region (but see for example, Ahrestani & Sankaran, 2016; Moe & Wegge, 1997; Ratnam et al., 2016, 2019; Sankaran, 2016), and experimental manipulative studies are largely lacking.

In subtropical monsoon grasslands, a large proportion of grasslands is burned every year, a practice that has long been an important element of grasslands in the region (Dinerstein, 1979; Ratnam et al., 2011, 2016; Sankaran, 2005, 2016). Burning of subtropical monsoon grasslands has been promoted as a cost‐effective method for grassland management in protected areas of the Cwa climate region (and also in Nepal). Therefore, we aimed at exploring the effect of fire on forage quality and associated pyric herbivory in an area that lies in the mesic region but receives a higher amount of mean annual precipitation than mesic savannas (*cf*. Ratnam et al., 2016; Ratnam et al., 2019).

Recent studies in pyric herbivory illustrate that burning can affect the movement of herbivores by attracting animals toward the burned areas due to regrowth after fire with higher concentrations of nutrients including nitrogen and phosphorus (Allred et al., 2011; Eby et al., 2014; Ratnam et al., 2016). Recently burned grasslands contain forage in lower quantity but of higher quality (Allred et al., 2011) and are used more heavily by smaller body‐sized ruminants than by larger body‐sized herbivores (Donaldson et al., 2018; Eby et al., 2014). Unlike larger body‐sized herbivores, small body‐sized ruminants have high metabolic requirements, thus, need high forage quality to meet their metabolic demands (Cromsigt et al., 2009; Gordon & Illius, 1996; Prins & Olff, 1998; van Langevelde et al., 2008). Thus, it can be argued that burning may not be an appropriate grassland management strategy used for herbivore conservation in areas with assemblages of different body‐sized grazing herbivores. Fire can create a homogeneous landscape (Archibald et al., 2005) which may not be suitable for the existing assemblage of different body‐sized grazing herbivores found in subtropical monsoon grassland in Nepal.

Burning interrupts the positive interaction between grazing and grazing lawns by diffusing grazing pressure away from grazing lawns. Grazing lawns are “nutrient hotspots” from where herbivores can maximize their energy intake (Thapa, de Jong, et al., 2021) and require frequent grazing to persist (Hempson et al., 2015; McNaughton, 1984). However, frequent fire in a productive system (due to high rainfall) but a low density of grazing herbivores (Ratnam et al., 2019) may cause grazing lawns to disappear. Consequently, tall and fast‐growing vegetation may re‐establish in the area, which is less beneficial to small and medium body‐sized grazers. The resultant vegetation is highly flammable in the dry season when the tall graminoids have dried up (Ratnam et al., 2019), and if fire is anthropogenically induced, often indiscriminate (van Langevelde, 2003a, 2003b).

**FIGURE 1:**
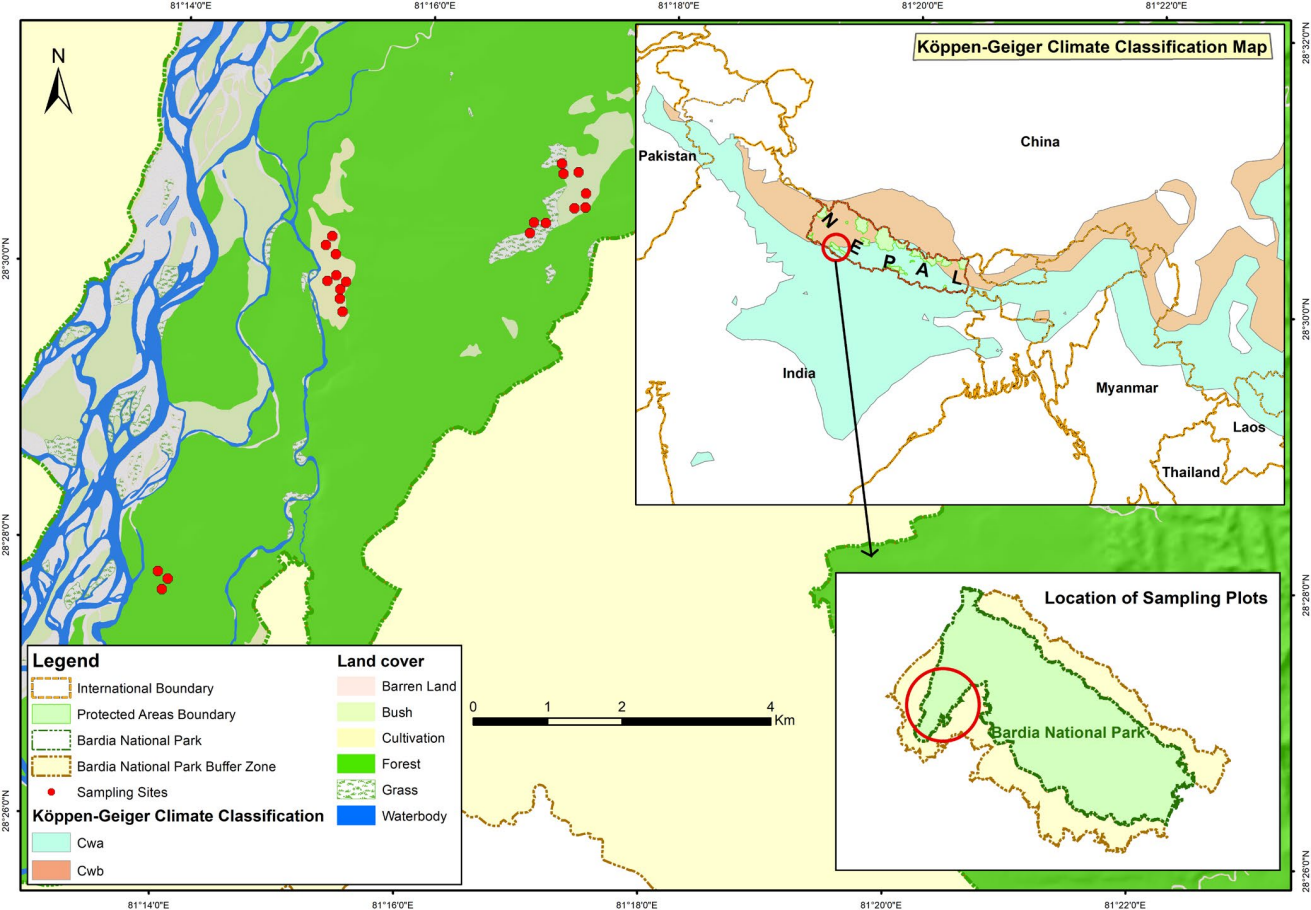
Locations of sampling plots within burned grasslands in Bardia National Park, Nepal. Bardia National Park lies within Terai Arc Landscape and has a Cwa‐climate according the Köppen classification (areas with light blue in the inset, top‐right)

Therefore, in order to use fire as a grassland management tool for the conservation and management of wild herbivores in subtropical grasslands, it is important to understand the dynamics of fire‐grazing interactions and factors driving pyric herbivory. Here, we report on the effect of a single fire event on the postfire regrowth quality, tested the quality of postfire regrowth as forage, and the resultant response of grazing herbivores to postfire regrowth in the subtropical grasslands of Bardia National Park (Bardia NP, West Nepal; Figure 1). First, we assessed the temporal pattern of postfire regrowth quality. Second, to examine whether the intensity of use is a function of postfire regrowth quality, we gauged the response of grazing herbivores to postfire regrowth over time. The intensity of use of the burned area by different body‐sized cervids may vary because of their body‐size (Cromsigt et al., 2009; Prins & Olff, 1998) and with respect to their feeding mode. Thus, we further assessed the intensity of use of postfire regrowth by the two most abundant cervids, chital (*Axis axis*), and swamp deer.

## 2 MATERIALS AND METHODS

### 2.1 Study area

We carried out our study in Bardia NP. The park lies in the Western Terai of Nepal (28°23′N, 81°30′E, elevation 100–1500 m.a.s.l., Figure 1). The Terai denotes the lowlands between the Ganges and the Himalayan foothills. The park covers an area of ~970 km^2^ and is surrounded by a buffer zone of ~500 km^2^. The national park is a “Level I—Tiger Conservation Unit” (Wikramanayake et al., 1998) and forms an essential component of the global tiger conservation strategy. The park and the surrounding buffer zone hold the second largest population of tiger in Nepal with an estimated density of ~5 individuals/100 km^2^ and an estimated prey density of ~78 km^−2^ (DNPWC & DFRS, 2018). The park is home to five cervids—from smaller to larger based on average adult body mass—northern red muntjac (*Muntiacus vaginalis*) with an average weight of ~30 kg, hog deer ~40 kg, chital ~50 kg, swamp deer ~150 kg, and sambar (*Rusa unicolor*) ~185 kg. Here, we classified the assemblage of these cervids as a mesofaunal deer community (Ahrestani & Sankaran, 2016). Chital is the most abundant and at the moment the primary prey species of the tiger in Bardia NP (Upadhyaya et al., 2018) with a reported density of ~50 deer.km^−2^ (DNPWC & DFRS, 2018). Muntjac and sambar are forest dwellers; are classified as browsers (Ahrestani & Sankaran, 2016) and are seen very rarely in the grasslands. Hence, the animals of interest for our study were chital—mixed feeder, swamp deer, and hog deer—categorized as grazers (Ahrestani & Sankaran, 2016).

**FIGURE 2:**
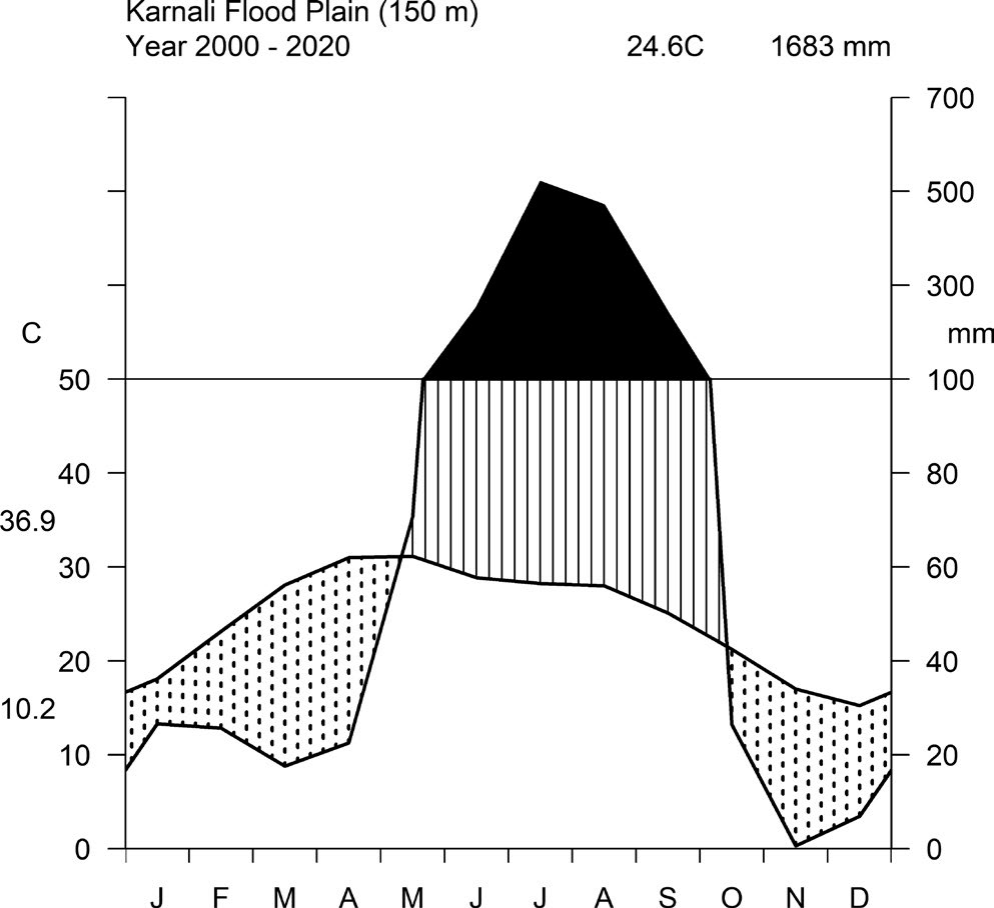
Walter ‐Lieth Climate diagram for Bardia NP. The diagram shows the mean rainfall and temperature for 2000–2020 (mean of the three weather stations Karnali‐Chisapani, Rajapur and Guleriya). The values on the upper right corner indicate the annual average temperature (24.6°C) and annual total rainfall (1683 mm). Area marked with dots indicates the dry‐period; area with vertical lines indicates the humid period; and area in solid black indicates the wet‐period (data source: Department of Meteorology and Hydrology, Nepal). March, April, and May are the peak dry‐period. This period is characterised by a high frequency of fires in the grasslands

The area has three distinct seasons: the very wet monsoon (June to September), the dry frost‐free winter (October to January), and the hot dry summer (February to May). The monthly mean temperature of the area ranges between 10°C in January and 45°C in June and the park receives a mean annual rainfall of ~1700 mm (Figure 2). According to the Köppen‐Geigen climate classification, the area falls within a Cwa climate: monsoon‐influenced humid subtropical climate (Chen and Chen, 2013), which extends from the Indus River to the South China Sea (Figure 1).

Bardia NP consists of diverse landscape elements ranging from riverine floodplain grasslands in the floodplains of the Karnali River and the Babai River; riverine forest; sal (*Shorea robusta* Gaertn. f.) forest; and mosaics of grasslands interspersed within the forests. The grasslands interspersed within the forests originated from human activities (i.e., land conversion) and are maintained either by grazing, grass harvesting, or by fire (Brown, 1998; Lehmkuhl, 1994; Peet, Watkinson, Bell, & Sharma, 1999; Wegge et al., 2000). *Imperata cylindrica* (L.), *Vetiveria zizanioides* (L.), *Narenga porphyrocoma* (Hance ex Trin.) Bor, and *Saccharum spontaneum* (Retz.) are the abundant graminoids in these grasslands (Peet, Watkinson, Bell, & Kattel, 1999; Thapa, de Jong, et al., 2021). The riverine floodplain grasslands along with the grasslands that are interspersed within forests represent the globally important ecoregion “Terai‐Duar Savanna and Grasslands” (Olson & Dinerstein, 2002).

### 2.2 Fire regime within Bardia NP

Based on freely available MODIS fire data, a total of 2013 fires were recorded within Bardia NP and its buffer zone by MODIS satellites from January 2010 to December 2020, out of which, around 75% fires were detected with more than 50% confidence. The maximum number of fire incidents occurred in the year 2016, followed by 2012 and 2019, respectively. The majority of fire incidents is observed in April (~60%), followed by May (~30%), which is consistent with previous studies (Ghimire et al., 2014; Thapa, Thapa, et al., 2021). Except natural barriers (e.g., rivers), only few fire breaks (fire‐line or forest roads) are constructed in the park to facilitate reducing the spread of surface fire. The forests in the Bardia NP (~70% of the area) are composed of subtropical species (e.g., sal) that shed large quantity of dry leaves during the winter, which results in a larger accumulation of fuel (Thapa, Thapa, et al., 2021). Likewise, grasslands are also composed of large quantities of litter (Ghimire et al., 2014; Thapa, de Jong, et al., 2021) that supports the spread of fire during the hot dry season.

Burning is a common grassland management practice that is being carried out by the park authority since its establishment (Peet, Watkinson, Bell, & Kattel, 1999). Local people also initiate fire to ensure good grass growth for next year thatch harvest, but it is not allowed by the park management. Thatch harvesting in grasslands of the protected areas is considered a means to pacify the park‐people relationship (Peet, 1997). Fires take place in more than 80% of the total park area (including forests) and almost all the grasslands are burned annually either by park staff or by local people after thatch harvest especially during March–May (Ghimire et al., 2014).

### 2.3 Experimental set‐up

Since 2018, ~75 ha (out of ~250 ha) of grasslands in the Karnali floodplain are under a long‐term experiment where grasses are being mowed frequently to establish the effect of cutting on nutrient concentrations in vegetation (Thapa et al., in prep.). These experimental areas were protected from fire and the remaining areas (~150 ha out of ~250 ha) were burned by park staff for grassland management. Within a week after such management fire at the end of March 2020, we established 60 m × 60 m plots (*n* = 21) randomly in three locations within these 150 ha of burned grassland patches (Figure 1). This enabled us to quantify changes in vegetation properties and resultant use by herbivores over a period after the fire. Four quadrats of 2 m × 2 m were permanently marked with pegs along the diagonal of each 60 m × 60 m plot at an equal distance of 20 m from where intensity of use (through pellet groups count) were recorded at regular 30‐day intervals for four months (end of April to end of July) from 30 days following the fire.

### 2.4 Vegetation characteristics

We collected postfire regrowth grass samples at regular 30‐day intervals for four months (from end of April to end of July) from the center of 60 m × 60 m plots. Postfire regrowth grass samples were clipped at ground level in a 0.36 m^2^ frame from each 60 m × 60 m plot and fresh weight was quantified using a digital weighing scale (with a capacity of 600 g and accuracy of 0.5 m; Brand: Equal [class II]) and estimated aboveground biomass. The clipped samples were hand‐sorted into green leaf, green stem, dead leaf, and dead stem and left to air dry for 5–6 days at ambient room temperature. The air‐dry weights of separated parts were recorded, and proportions of green leaf and dead parts were determined. The separated grass parts were mixed again and packed in a paper bag for chemical analyses. Mean grass height was calculated for each plot by measuring to the nearest centimeter at three points within each 2 m × 2 m quadrat while recording the pellet groups. The same observer collected grass samples, measured the height, and counted pellet groups.

Air‐dried grass samples were oven‐dried for 48 h at 60°C to prevent caramelization, grinded, and sieved over a 2 mm sieve for chemical analyses. Nitrogen (N) was determined by a semi‐micro Kjeldahl method in dry‐block digester; phosphorus (P) by tissue digestion in block digester (AOAC, 1990); neutral detergent fiber (NDF) and acid detergent fiber (ADF) by the method described by Van Soest (1982); and silica by gravimetric method (AOAC, 1990). N:P ratios in plant tissues were calculated to test for nutrient limitation of vegetation growth (e.g., Koerselman & Meuleman, 1996; Ludwig et al., 2001). Nutrient concentrations were measured as percentage dry matter (% DM).

We estimated physical [biomass (g.m^−2^), height (m), bulk density (biomass × height − g.m^−3^), proportion of green leaf, and proportion of dead parts] and chemical [crude protein (CP; calculated as 6.25 × percentage nitrogen), phosphorus, NDF, ADF, and silica] parameters from postfire regrowth grass samples to examine the effect of fire on postfire regrowth quality.

### 2.5 Herbivore use of postfire regrowth

We used pellet groups as a proxy to measure the intensity of use for grazing (*cf*. Putman, 1984; Kohn & Wayne, 1997; Sánchez‐Rojas & Gallina, 2000; Hemami et al., 2005; Skarin, 2007; Hegland et al., 2010) by the mesofaunal deer assemblage (chital, swamp deer, and hog deer) in relation to the postfire regrowth. We observed deer very rarely lie down and rest in these grasslands, so we assumed that pellet density mainly reflects the intensity of use. Pellet groups were recorded by species based on individual pellet morphology which we were able to do after collecting droppings from deer sighted to defaecate. Pellets of hog deer are rounded more like pigeon‐pea shape; pellets of swamp deer are big cylindrical and flat on both ends, whereas pellets of chital are narrow, long cylindrical, smaller than swamp deer, and tapered at one end (Ahrestani et al., 2018; pers.obs.). Yet, we are very aware of potential misidentification between ungulate dropping (see, e.g., Spitzer et al., 2019). We made our field team familiar with the different morphological features of pellets of the three species, and hence, reduced the possible error of misidentification.

Pellet groups were counted in the 2 m × 2 m quadrats (e.g., Supartono et al., 2021) at regular 30‐day intervals for fourth months following the fire. Pellet groups of which the center fell outside the boundary line of 2 m × 2 m quadrats were not included in the count and only pellet groups containing five or more pellets were recorded to prevent counting droppings of deer merely passing through. We removed all pellets from each quadrat to avoid recounting during the subsequent surveys. For each plot, we summed the pellets of individual species at the plot level and used them for statistical analysis.

### 2.6 Data analysis

All statistical analyses were computed using the R‐program, version 4.1.0. (R Core Team, 2021). As there may be a spatial autocorrelation between the datapoints due to the spatial setup of the research design, we checked for spatial dependency of the response variable (especially pellet groups) with respect to places (plots in our case) by calculating Moran's I (Salima & Bellefon, 2018), and plotting the Moran's scatterplot using the “spdep” package (Bivand & Wong, 2018). We found that there is no/hardly any autocorrelation present in the data (Moran's I = 0.18; Figure S4). The Moran's I index ranges from −1 (strong negative spatial autocorrelation) to 1 (strong positive spatial autocorrelation), and a value of zero indicates no spatial autocorrelation (Salima & Bellefon, 2018).

Changes in postfire regrowth physical and chemical properties with respect to time after fire, namely, 30 to 120 days after fire, were estimated to depict the effects of fire on forage quality. A Kruskal–Wallis test using the “kruskal.test” function followed by multiple comparisons using the “kruskal” function (“agricola” package) was performed (due to non‐normality nature of the data) to compare the differences in postfire regrowth grass height measured at 30, 60, 90, and 120 days after fire, respectively. Linear model analyses were performed for N:P ratios and log‐transformed variables (biomass and bulk density) using the “lm” function to estimate the changes in N:P ratio, biomass, and bulk density in postfire regrowth with respect to different sampling instances. Beta regression was performed for proportion and percentage data (*viz*., CP, phosphorus, NDF, ADF, proportion of green leaf and dead parts) using the “betareg” function [“betareg” package (Cribari‐Neto & Zeileis, 2010)] to measure the changes in the parameters in the postfire regrowth with respect to time since fire. The main effects of the beta regression models were evaluated by Type II Wald chi‐square (χ^2^) tests using the “Anova” function [“car” package (Fox & Weisberg, 2018)]. Post hoc multiple comparisons tests were performed using the “emmeans” function (“emmeans” package) and the “cld” function [“multicomp” package (Hothorn et al., 2008)] after linear and beta regressions.

To assess the intensity of use (grazing) by the mesofaunal deer assemblage in relation to postfire regrowth, we performed multiple tests using pellet group count data as a response variable. We tested two statistical models to assess the effects of the postfire regrowth grass height on (i) intensity of use of burned areas for grazing by Generalized Linear Model (GLM) with Poisson distribution; and (ii) vegetation CP levels by GLM with gamma distribution. Likewise, we also tested the effect of CP levels on the intensity of use by GLM with a Poisson distribution. In addition, we also tested the effect of the postfire regrowth biomass on the intensity of use by GLM with a Poisson distribution. The GLM analyses were performed using the “glm” function. R‐squared values for the GLM models were calculated using the “rsq” function (type = KL, “rsq” package). Wald test was performed to test for the significance of the coefficients of GLMs using the “wald.test” function (“mdscore” package). We used GLM because of its flexibility and its ability to handle a larger class of distributions for the response variables (Guisan et al., 2002; Guisan & Harrell, 2000; O’Hara & Kotze, 2010; Okamura et al., 2012; Warton et al., 2016). For each GLM with a Poisson distribution model, the residuals were plotted against fitted values (Coelho et al., 2020) and checked for over/underdispersions (Figures S5–S7). Likewise, we inspected the correlations between the variables using a correlation map (Figure S8) using the “ggcorplot” package (Kassambara, 2019). We have not used highly correlated variables (e.g., height and biomass, Pearson correlation coefficient: *r* = .80) together in a single model, for that reason, we did not have to account for collinearity.

We binned the grass heights into six classes (*viz*., 0–0.20 m; 0.21–0.40 m; 0.41–0.60 m; 0.61–0.80 m; 0–81–1.00 m; and >1.00 m) to identify which grass height classes are favored by the deer. For each height category, we reassigned “1” for the presence of pellet group/s and “0” for the absence of pellet group of either chital or swamp deer. We used a Chi‐square (χ^2^) test to compare the observed pellet group frequency of chital and swamp deer within the different grass height classes. We further calculated the proportion of observed and expected pellet groups per height class for chital and swamp deer and presented it in a graph to assess which grass height classes were preferred by the two deer species. We did this to assess whether or not a differential use of the burned area by two abundant cervids (chital and swamp deer) took place because these two species have a distinct morphology and feeding behavior. We expect their physiological needs to be different because of their differing body sizes (Cromsigt et al., 2009; Prins & Olff, 1998). We did not include hog deer for analyses because of an insufficient amount of data due to their relative rarity.

Descriptive statistics (e.g., mean with 95% CI) for proportion data (proportion of green leaf and proportion of dead parts), and chemical parameters (CP, phosphorus, NDF, ADF, and silica) were calculated with arcsine transformed data and back‐transformed for interpretation. All graphs were prepared using ggplot2 (Wickham, 2021).

## 3 RESULTS

### 3.1 Postfire regrowth chemical properties

**FIGURE 3:**
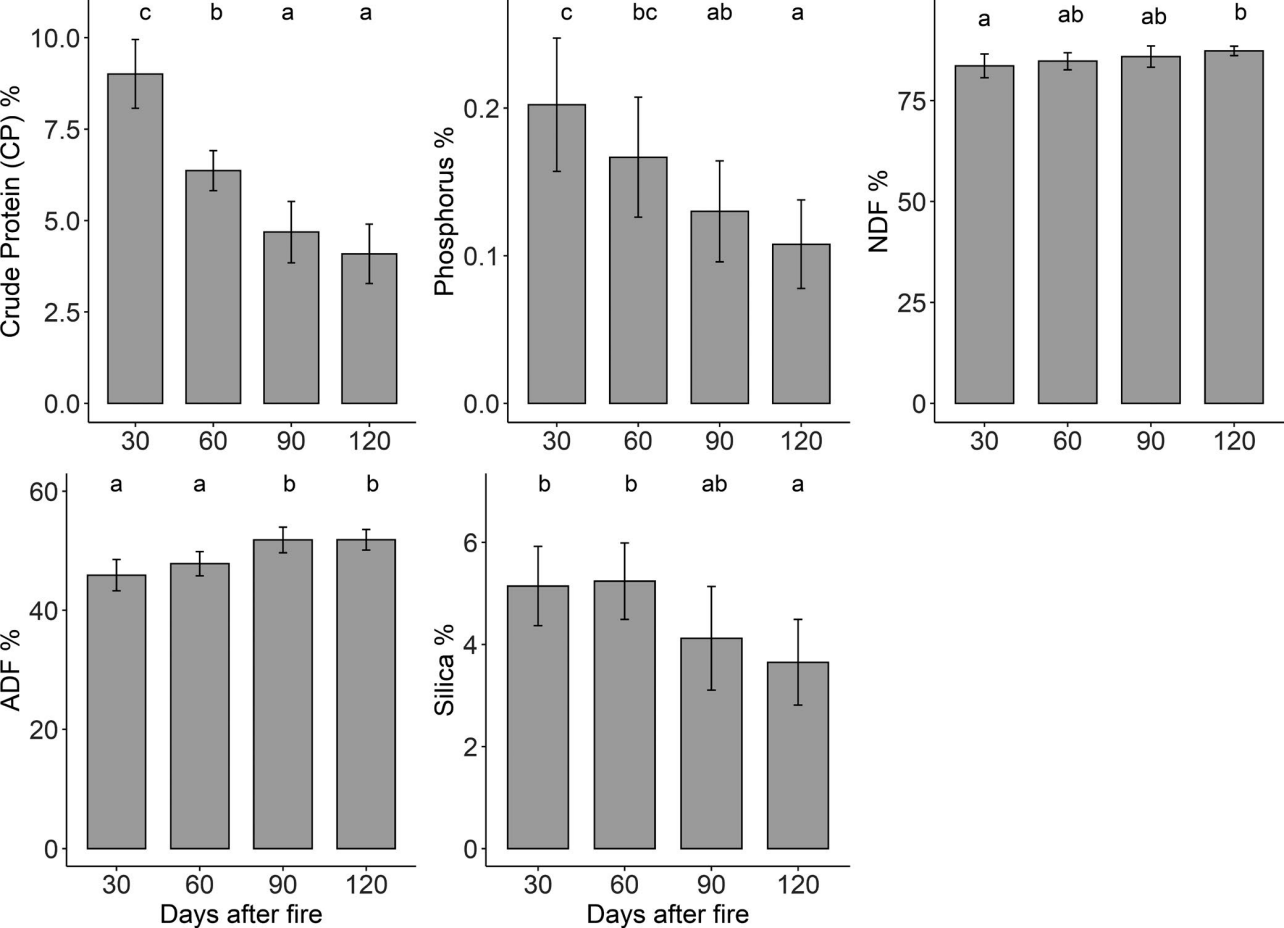
Chemical parameters (% DM) in post‐fire regrowth grass tissues sampled from subtropical grasslands in Bardia NP, Nepal at different time intervals after fire. Bar graphs show mean (±95% confidence interval—CI). Scale of y‐axis varies with parameters indicated in the y‐axis. Letters above each bar indicates a significant difference at alpha = 0.05, tested by estimated marginal means after beta regression. Group that share same letter are not significantly different from each other

Forage nutritive values were dependent upon time since fire. Significant differences were found for important chemical parameters (*viz*, CP, phosphorus, NDF, ADF, and silica) in grass tissues while comparing for different times after fire, indicating a clear temporal sequence of differences (Figure 3). We found significantly higher concentration of crude protein, phosphorus, and silica in grass tissues at 30 days after fire than at other sampling instances (Figure 3). We observed strong and significant reductions in crude protein (mean value 9.1 to 4.1 [55% decrease]; Type II Wald chi‐square χ^2^ = 116.64, *df* = 3, *p* < .001), phosphorus (mean value 0.2 to 0.11 [45% decrease]; χ^2^ = 22.59, *df* = 3, *p* < .001) and silica (mean value 5.2 to 3.6 [31% decrease]; χ^2^ = 14.84, *df* = 3, *p* < .001) in forage samples collected during different time intervals, that is, from 30 days to 120 days after fire (Figure 3). Likewise, we found increased NDF and ADF levels (Figure 3) in grass samples from 30 days to 120 days after fire (χ^2^ = 10.35, *df* = 3, *p* = .016; and χ^2^ = 34.96, *df* = 3, *p* < .001). The N:P ratio did not differ significantly between the days after fire (linear model *F* = 0.48, *df* = 3, *p* = .690; Figure S1a); but the N:P ratio was below 10 in the postfire regrowth in each sampling instance after fire (Figure S1b).

### 3.2 Postfire regrowth physical properties

Postfire regrowth height and biomass were significantly lower in the first sampling instance (i.e., 30 days after fire) than at the other sampling instances (Kruskal–Wallis, Χ^2^ = 65.261, *df* = 3, *p* < .001, and linear model, *F* = 101, *df* = 3, *p* < .001, respectively). In addition, plant height and biomass showed a significant increase with time since fire (Table 1). Bulk density was significantly higher in the first sampling instance (i.e., 30 days after fire) when compared with other sampling instances (linear model, *F* = 14.46, *df* = 3, *p* < .001), while the proportion of green leaf was highest in the second sampling instance, that is, 60 days after fire (Type II Wald chi‐square χ^2^ = 31.33, *df* = 3, *p* < .001, Table 1). Likewise, proportion of dead parts in the postfire regrowth samples was significantly higher in 30 days after fire (Type II Wald chi‐square χ^2^ = 45.93, *df* = 3, *p* < .001) and decreased with time since fire (Table 1).

**TABLE 1:** Mean with 95% CI for vegetation physical properties collected during four different time after fire from the grassland of Bardia NP

Vegetation physical properties	Days after fire				Method
	30 days	60 days	90 days	120 days	
Height (m)	0.17 (0.14–0.19)^a^	0.32 (0.26–0.38)^b^	0.82 (0.71–0.92)^c^	0.98 (0.85–1.11)^d^	Multiple comparison Fisher's least significant difference after Kruskal–Wallis test
Biomass (g.m^−2^)	171 (142–206)^a^	194 (172–221)^a^	388 (345–446)^b^	713 (645–788)^c^	Multiple comparison with estimated marginal means after linear model
Bulk density (g.m^−3^)	1075 (863–1339)^c^	658 (544–812)^ab^	497 (445–601)^a^	757 (665–897)^b^	Multiple comparison with estimated marginal means after linear model
Proportion of green leaf	0.45 (0.39–0.50)^a^	0.62 (0.57–0.67)^b^	0.53 (0.49–0.58)^a^	0.48 (0.43–0.52)^b^	Multiple comparison with estimated marginal means after beta regression
Proportion of dead parts	0.48 (0.40–0.56)^b^	0.31 (0.26–0.36)^a^	0.25 (0.22–0.29)^a^	0.24 (0.21–0.27)^a^	Multiple comparison with estimated marginal means after beta regression

Note

Letters in the cells indicate significant difference at alpha = 0.05. Groups that share the same letter are not significantly different from each other.

### 3.3 Response of mesofaunal deer to postfire regrowth

**FIGURE 4:**
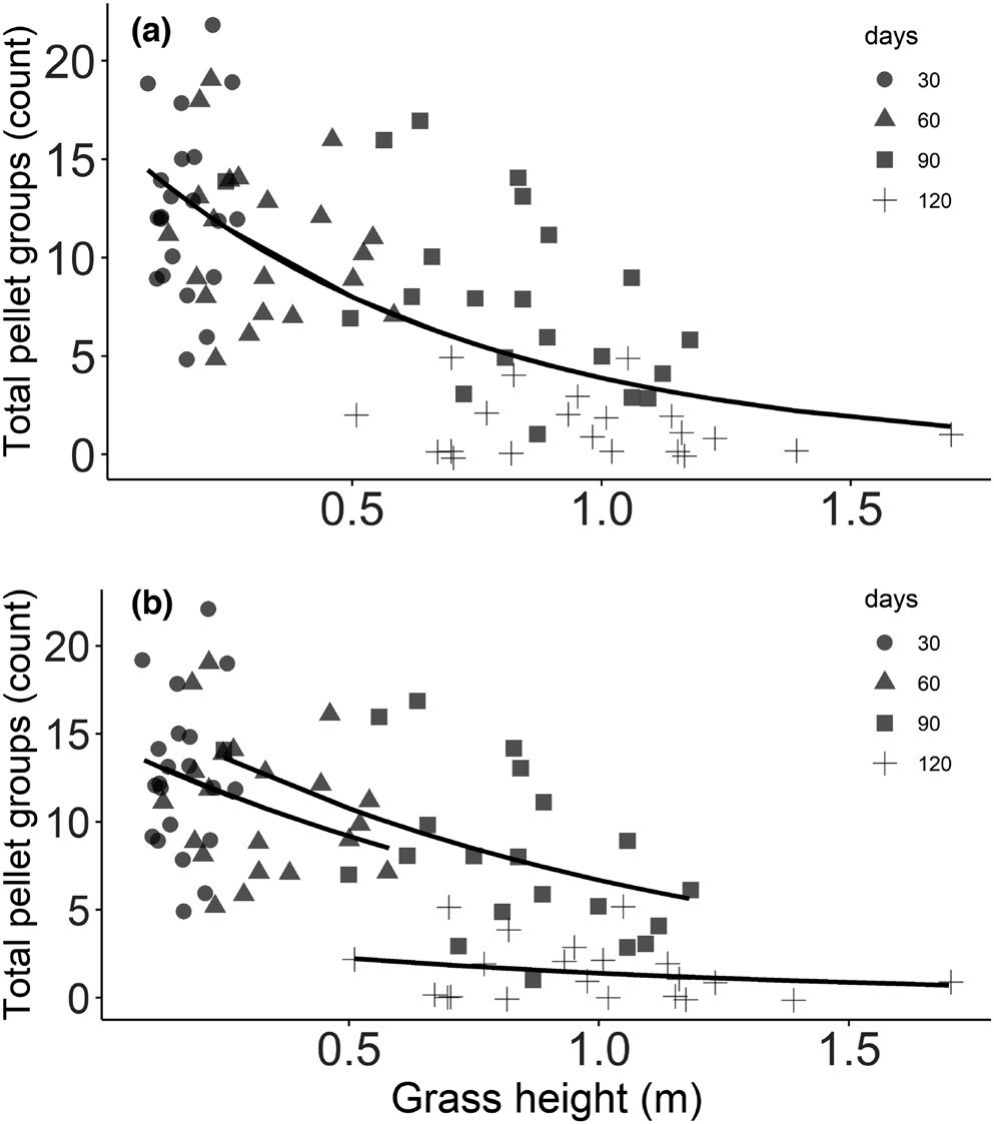
(a) Density of total pellet groups (proxy for the herbivore indicative of intensity of use of an area) in relation to grass height (cm) and time since fire (i.e., 30, 60, 90 and 120 days) in the burned grassland areas in Bardia NP. A: The equation of the line, generated by GLM with Poisson distribution, is log(μ) = 2.8 − 1.44 × grass height; *R* ^2^ = .43. in which μ stands for pellet density. (b) Same data as in panel A but with ‘grass height’ and ‘days since fire’ as covariates in the model. The equation for the lines are (i) for 30 days is: log(μ) = 2.688 − 0.95 × grass height; (ii) for 60 days: log(μ) = 2.69 − 0.95 × grass height; (iii) for 90 days: log(μ) = 2.85 − 0.95 × grass height; and (iv) for 120 days: log(μ) = 1.29 − 0.95 × grass height. μ stands for pellet density

**FIGURE 5:**
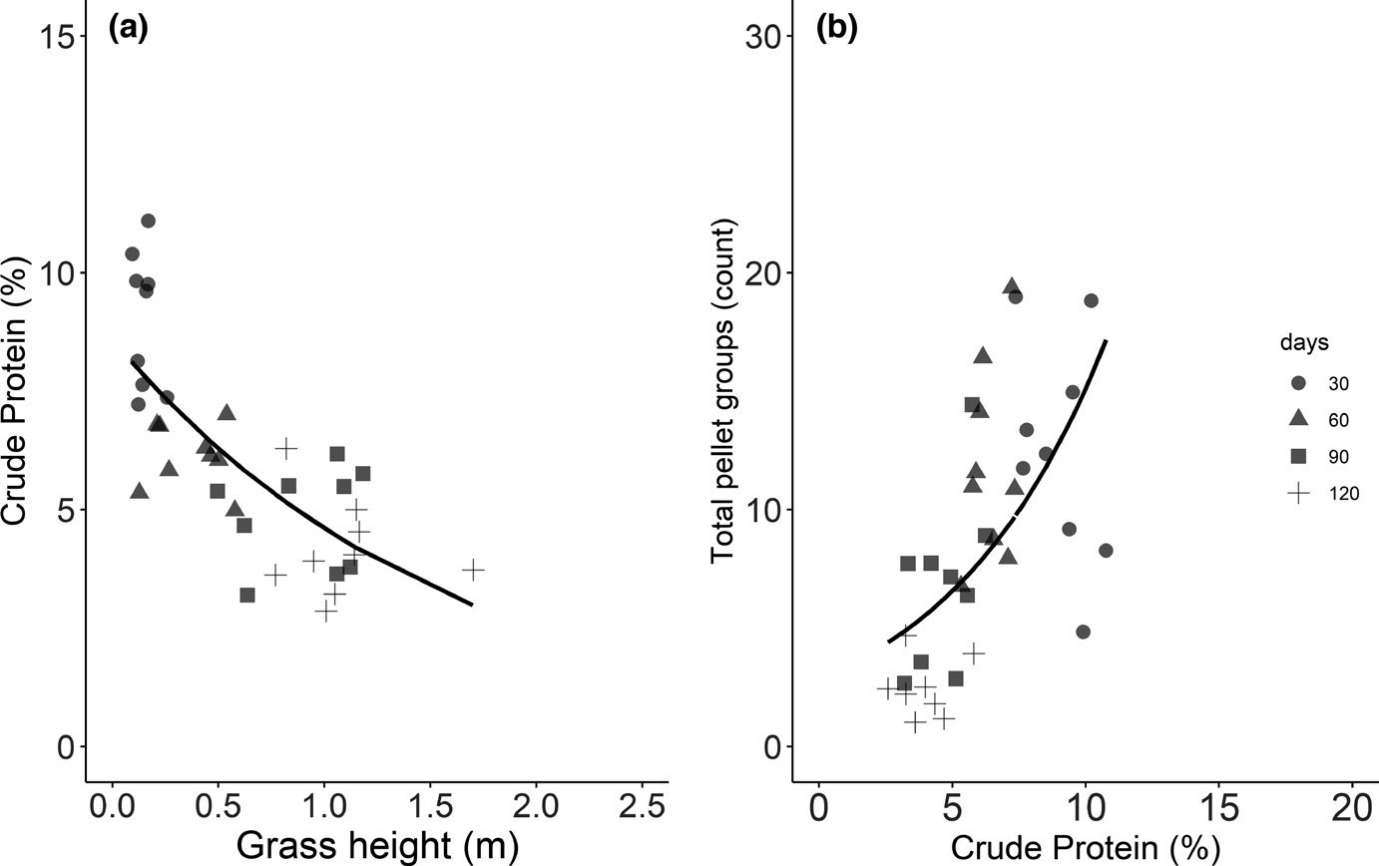
Panel (a) Relationship of post‐fire regrowth grass crude protein (%) levels to grass height (m) and Panel (b): total pellet groups (as proxy for the herbivore foraging intensity) to grass crude protein (%) recorded along the time since fire (i.e., 30, 60, 90 and 120 days) from the burned grassland areas in Bardia NP. The equation of the line for A is log(μ) = 2.15 − 0.62 × grass height; *R* ^2^ = .56 (generated by GLM with Gamma distribution), and that for B is log(μ) = 1.05 + 0.17 × crude protein; *R* ^2^ = .31 (generated by GLM with Poisson distribution). μ stands for pellet density

The effect of fire on grazing herbivores in the grasslands of Bardia NP was highest in the first sampling instance and decreased with time since fire. The intensity of use (based on pellet groups count) of postfire regrowth by the mesofaunal deer assemblage (especially by chital, swamp deer, and hog deer) showed a negative association with grass height (Wald test W = 140.49, *p* < .001; Figure 4) and biomass (Wald test W = 157.46, *p* < .001; Figure S2). Likewise, the level of crude protein in grass tissues decreased with increasing grass height (Wald test W = 45.22, *p* < .001; Figure 5a), and the intensity of use by mesofaunal deer was higher in the areas with higher levels of crude protein (Wald test W = 42.91, *p* < .001; Figure 5b).

**FIGURE 6:**
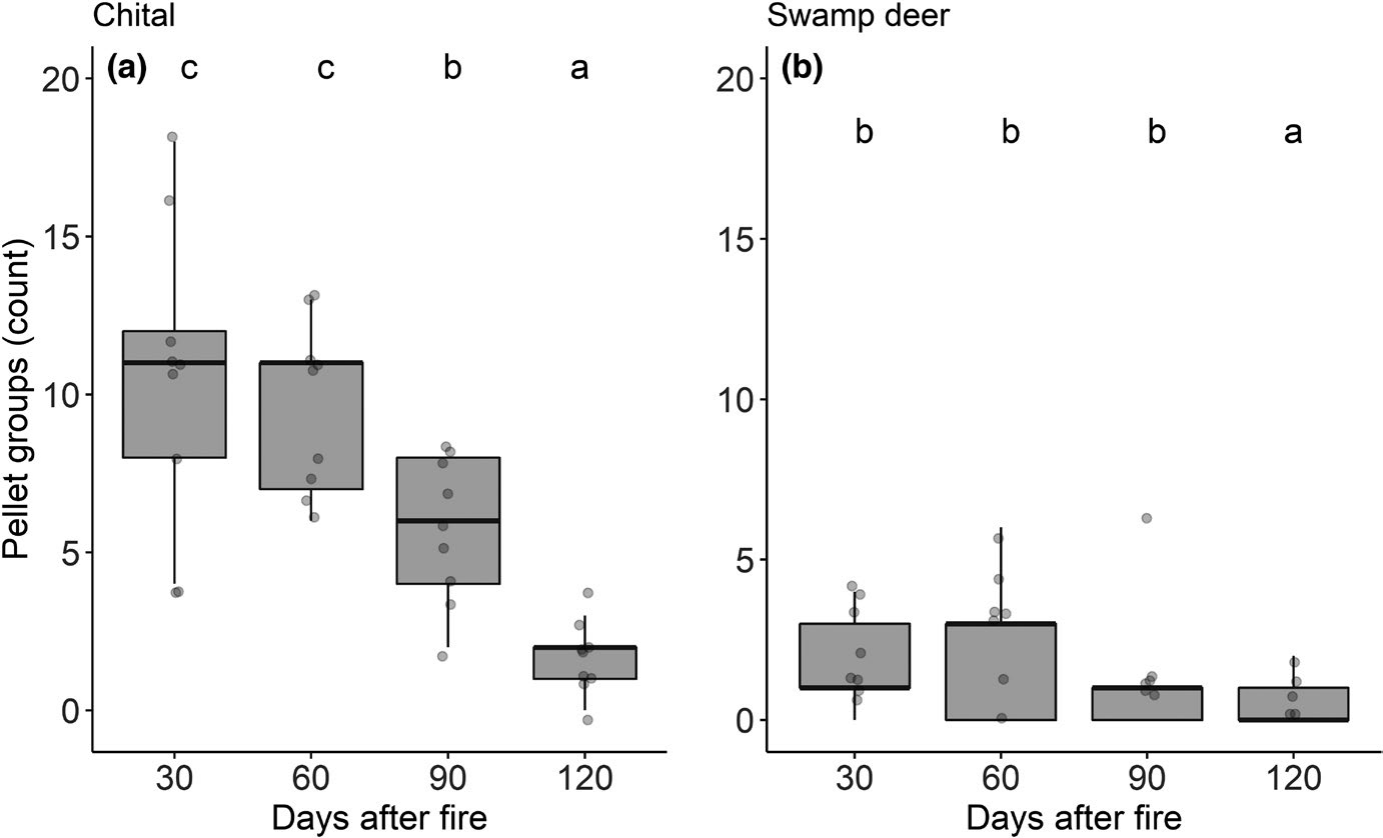
Pellet groups recorded from different periods after fire for (a) chital and (b) swamp deer. Letter above each boxplot show significant difference at alpha = 0.05, tested by estimated marginal means after GLM with Poisson distribution. Group that share same letter are not significantly different from each other

The intensity of use by chital to postfire regrowth differed significantly over time since fire, with higher intensity of use at 30 days after fire (Figure 6a; Table 2). We did not find a significant difference in the intensity of use by swamp deer until 90 days after fire. However, the level of use by swamp deer decreased significantly during the fourth sampling period (120 days after fire; Figure 6b; Table 2). The intensity of use by both chital and swamp deer was higher when postfire regrowth grass height was below 40 cm and lower after 60 days postfire (when the grass height exceeded 40 cm; Chi‐square, χ^2^ = 12.737, *p* = .026; χ^2^ = 13.36, *p* = .030, respectively, Figure S3).

**TABLE 2:** Statistical parameters (estimated value for coefficient; SE, standard error for estimate of coefficient; z value; and *p* value) from different time period after fire for chital and swamp deer

	Estimate	SE	z‐value	*p*‐value
Chital				
Intercept	2.397	0.06	36.44	<.001
60 days after fire	−0.201	0.09	−2.05	.041
90 days after fire	−0.552	0.11	−5.07	<.001
120 days after fire	−2.184	0.21	−10.56	<.001
Swamp deer				
Intercept	0.452	0.17	2.59	.009
60 days after fire	0.217	0.23	0.93	.353
90 days after fire	0.141	0.23	0.59	.553
120 days after fire	−1.887	0.48	−3.93	<.001

Note

Model parameters include pellet group count of either chital or swamp deer and days since fire (days) fitted with GLM Poisson distribution.

## 4 DISCUSSION

By quantifying physical and chemical parameters of postfire regrowth on grasslands of Bardia NP over time for four months since fire, we were able to assess the primary factors explaining the aggregation of grazing herbivores in burned grasslands and better understand the temporal extent of pyric herbivory in the subtropical grasslands under control of the Cwa monsoon climate. Such a temporal effect of fire on postfire regrowth quality and associated pyric herbivory was already documented for African savannas (Archibald & Bond, 2004; Archibald et al., 2005; Archibald & Hempson, 2016; Donaldson et al., 2018; Eby et al., 2014; Klop et al., 2007; Van de Vijver et al., 1999) and North American prairies (Allred et al., 2011; Fuhlendorf et al., 2009; Ratnam et al., 2016; Veach et al., 2014). Moreover, we showed that time since fire is indeed a critical determinant of the postfire regrowth quality and associated pyric herbivory in subtropical monsoon grasslands that lies outside the average annual rainfall range of mesic savannas (*cf*. Sankaran et al., 2005; Ratnam et al., 2016, 2019). Only few studies on pyric herbivory (e.g., Moe & Wegge, 1997; Sankaran, 2016) are available from this region. Furthermore, we showed that the pattern of usage of burned areas by two cervids *viz*., chital and swamp deer differ significantly with respect to time since fire. Thus, our study adds important insights on pyric herbivory from this region which can be extended to a much larger area in Asia within the Cwa climate.

### 4.1 Postfire regrowth quality as a driver for pyric herbivory

We found a distinct temporal pattern of forage nutritive value of grasslands of Bardia NP induced by fire. Our results depicted that the postfire regrowth grass quality was higher immediately after fire (i.e., 30 days after fire) but decreased over time. Both physical and chemical properties of postfire regrowth vegetation in the first weeks (i.e., 30 days after fire) resulted in a higher food value for grazing herbivores when compared to later sampling instances (i.e., 60, 90, and 120 days after fire; Table 1 and Figure 3). Fire increased forage crude protein (CP) and phosphorus (P) concentrations (refer to Figure 3) to the level that is required by mesofaunal deer (especially for chital) for maintenance and reproduction, but not for lactation (Thapa, de Jong, et al., 2021). But this increased forage CP and phosphorus is available only for a short period (not more than 60 days). Based on the known allometric relationship (Ahrestani et al., 2012; Prins & Van Langevelde, 2008), the nutritional requirements of deer differ due to differences in body size and also with respect to physiological needs—maintenance, reproduction, and lactation. Peak parturition timing for chital is between February and April (Thapa, de Jong, et al., 2021), for swamp deer, it is late September (Dinerstein, 1980), and that for hog deer is March through April (Dhungel & O’Gara, 1991). Chital and hog deer may benefit briefly during the lactation period due to availability of higher levels of nitrogen and phosphorus in the postfire regrowth vegetation. However, swamp deer may have to rely on nutrient‐poor matured tall grasses even in the lactation period, a period when the animal has higher demand of nutrition to improve her lactation ability and milk quality (Ahrestani et al., 2012).

The CP concentration in the postfire regrowth grass tissues collected after 30 days since fire was comparable to the levels reported from grazing lawns but higher than the levels reported from unburned tall grass samples. Thapa, de Jong, et al. (2021) reported that the CP levels in green leaves from grasslands of Bardia NP ranged between 8.9% and 10.0% for grazing lawns and for unburned tall grasses it ranged between 7.1% and 8.3%. The level of CP in postfire regrowth grasses after 60 days since fire, ranged between 5.8% and 6.9%. This is lower than the CP level found in green leaves from unburned tall grasses (Thapa, de Jong, et al., 2021), indicating that the availability of a higher level of nitrogen from postfire regrowth does not last long (not even for 60 days after fire). Similar findings of fire‐induced nutritional increase for a short period have been reported for African savannas (Allred et al., 2011; Archibald & Bond, 2004; Eby et al., 2014; Van de Vijver et al., 1999). Following fire, forage quality declined when postfire regrowth gained height and biomass (Table 1), affecting the intensity of use by mesofaunal deer (Figures 4 and 5, and Figure S2). Both nutritive value and digestibility are inversely related to grass height and biomass; as grasses mature, height and biomass increase (Table 1), whereas quality (e.g., CP; Figure 5a) and digestibility decreases (Anderson et al., 2007; Thapa, de Jong, et al., 2021; Van Soest, 1994).

Pyric herbivory is thus advantageous for mesofaunal deer, in particular during the hot dry season, as it stimulates fresh, high‐quality new growth (albeit only up to two months). Yet, it appeared that the abundant mesofaunal deer in the grasslands of Bardia NP were not able to maintain the grasses in a short grazing lawn state [the desired state to realize high energy gain for grazing herbivores (Thapa, de Jong, et al., 2021)]. As a result, nutrients—especially nitrogen—diminished over time after fire and with grass height (see Table 1; Figures 3 and 5a). It appears that the existing density of the grazing herbivores in these grasslands could not exert sufficient grazing pressure to culminate in a herbivore‐driven system (Smit & Coetsee, 2019)—a desired positive effect of pyric herbivory, or maybe because of the “magnet effect” caused by the spatial extent of fire as explained by Archibald et al. (2005). Besides, studies from African savannas indicated that a high fire frequency leads to decreased foliar nitrogen and phosphorus values (Anderson et al., 2007) and also, a loss of nitrogen from the system, leading to a decrease in productivity (Van de Vijver et al., 1999). The grasslands in Bardia NP, where our study was executed, are burned annually (Peet, Watkinson, Bell, & Sharma, 1999) and our result also showed that the postfire regrowth was N‐limited for biomass production as the N:P ratio (such as estimated from foliar N and P) was <10 (see Figure S1).

### 4.2 Postfire regrowth and level of use by two cervids

Our findings showed that the mesofaunal deer utilized the burned areas extensively for a short period (up to 60 days after fire) until the area contained short grasses with lower levels of biomass (Figure 4 and Figure S2) and a higher level of protein (Figure 5b). The findings support research related to the forage maturation hypothesis (see for detail Fryxell, 1991; Olff et al., 2002; Prins & Olff, 1998; Ratnam et al., 2016; Wilmshurst et al., 2000) which emphasize that grazing herbivores select the foraging ground containing low to intermediate biomass to maximize their daily rate of energy gain.

Chital and swamp deer showed different responses to the postfire regrowth in Bardia NP (Figure 6 and Table 2). The intensity of use by chital, having a body mass ~50 kg, to burned grasslands differed significantly with time since fire with a higher level of use up to 60 days after fire (Figure 6a, Table 2), while swamp deer, having a body mass ~150 kg, did not show a clear pattern of use up to 90 days since fire (Figure 6b and Table 2). These differences may be explained by the energy requirement with respect to body mass (Illius & Gordon, 1992; Olff et al., 2002; Prins & Olff, 1998), as well as by the feeding mode of these two cervids. Chital is a mixed feeder and feeds primarily on grasses and switches to browse when grass quality declines and is considered more selective while cropping grass parts (Ahrestani & Sankaran, 2016). Swamp deer, on the other hand, is a grazer and feeds primarily on grasses and aquatic weeds (Ahrestani & Sankaran, 2016), and can digest taller and more coarse grasses than chital.

Our findings did not portray any evidence of differential use of burned areas to grass height (Figure S3). Both species preferred to graze in grassland with a grass height lower than 40 cm. This is in contrast to studies that suggest resource‐use partitioning through grass height (Cromsigt & Olff, 2006; Mandlate et al., 2019). Along with short grasses with higher quality, one could argue that an increased aggregation of mesofauna deer in the burned areas could be attributed to the reduced predation risk as a result of increased visibility created by burning [as indicated by studies from other parts of the world, e.g., Klop et al. (2007)]. In addition, environmental variables (*viz*., distance to forest, water, and roads) associated with burned areas are important attributes that are likely to influence the foraging behavior and space use by herbivores (Allred et al., 2011; Cherry et al., 2017; Marchand et al., 2017).

### 4.3 Management implications

The most dominant graminoids viz., *Imperata cylindrica* (L.), *Vetiveria zizanioides* (L.), *Narenga porphyrocoma* (Hance ex Trin.) Bor, and *Saccharum spontaneum* (Retz.) in Bardia NP (Thapa, de Jong, et al., 2021) get moribund during the cool dry winter and are grazed less by the existing herbivores unless the dry aboveground biomass is removed either by burning or cutting (Moe & Wegge, 1997; Peet, Watkinson, Bell, & Kattel, 1999; Wegge et al., 2006). The cool dry winter and hot dry summer seasons are a nutrient bottleneck period (Ahrestani et al., 2011) and during this time, a new flush of grasses becomes the valuable food source for herbivores. Given the widespread use of fire as a cost‐effective grassland management tool in subtropical monsoon grasslands in the Cwa climate region, it is important to realize that the positive benefit of a single event fire for the conservation of large herbivores is time specific, as the effect of fire on forage quality perhaps lasts for 60 days only. Chital is a mixed feeder, whereas swamp deer is a grazer and their level of energy requirements is different with respect to their body size. Hence, large‐scale single event fires may not fulfil the nutritional requirements of all mesofaunal deer. Furthermore, larger scale fires promote a uniform grazing environment where grazers are dispersed widely, resulting in a decreased grazing pressure in existing grazing lawns (Archibald & Bond, 2004; Archibald et al., 2005), and a fast increase in unpalatable grass biomass (Thapa, de Jong, et al., 2021).

Our results showed that biomass and height increased significantly with time resulting in the limited use of the burned areas after 60 days since fire. This indicated that the existing density of the mesofaunal deer assemblage in the Bardia NP was not able to maintain the grass height to the desired short state after fire occurrence. Furthermore, the grazing systems in the Cwa climate region is constrained by nitrogen for grass growth, as the N:P ratio estimated from foliar N and P was <10 (Koerselman & Meuleman, 1996) and phosphorus for herbivore productivity (Thapa, de Jong, et al., 2021). Indeed, it is not a management goal to increase the enormous production of grasses in this monsoon grassland, rather, the stated management goal of Bardia NP is to be a safe habitat for the endangered tiger population for which sufficient prey must be available.

In this respect, we recommend considering a spatiotemporal manipulation of fire to reinforce the grazing feedback for culminating in from fire‐dominated to herbivore‐dominated state. It is likely that the burned mosaics of grassland patches are intensively grazed resulting in the establishment of grazing lawns (Hempson et al., 2015; Thapa, de Jong, et al., 2021). Hence, a series of fires, staggered over time, may thus yield for the longest possible period a good food supply during the nutrient bottleneck months (cool dry winter and hot dry summer seasons) till the next growing season (starting with monsoon June through September), thus facilitating maximum survival for the deer that are to be preyed upon by the tiger.

## CONFLICT OF INTEREST

No conflict of interest.

## AUTHOR CONTRIBUTIONS

**Shyam Kumar Thapa:** Conceptualization (equal); Formal analysis (lead); Methodology (lead); Project administration (lead); Writing – original draft (lead); Writing – review & editing (equal). **Joost F. de Jong:** Conceptualization (equal); Formal analysis (equal); Methodology (equal); Supervision (equal); Writing – review & editing (equal). **Anouschka R. Hof:** Conceptualization (equal); Supervision (equal); Writing – review & editing (equal). **Naresh Subedi:** Conceptualization (supporting); Funding acquisition (lead); Methodology (equal); Supervision (equal); Writing – review & editing (equal). **Laxmi Raj Joshi:** Data curation (equal); Formal analysis (supporting); Methodology (supporting); Writing – original draft (supporting); Writing – review & editing (equal). **Herbert H. T. Prins:** Conceptualization (lead); Funding acquisition (lead); Supervision (lead); Writing – review & editing (equal).

### OPEN RESEARCH BADGES

This article has been awarded Open Data, Open Materials Badges. All materials and data are publicly accessible via the Open Science Framework at [https://doi.org/10.5061/dryad.2jm63xsqz].

## Supporting information

Fig S1

Fig S2

Fig S3

Fig S4

Fig S5

Fig S6

Fig S7

Fig S8

## Data Availability

The data that support the findings of this study are openly available in: Dryad, Dataset https://doi.org/10.5061/dryad.2jm63xsqz.
