# Supplementary figures and images for: Fire and forage quality: Postfire regrowth quality and pyric herbivory in subtropical grasslands of Nepal
Source: Ecol Evol. 2022 Apr 13;12(4):e8794. doi: 10.1002/ece3.8794 (PMC9006228; doi:10.1002/ece3.8794)

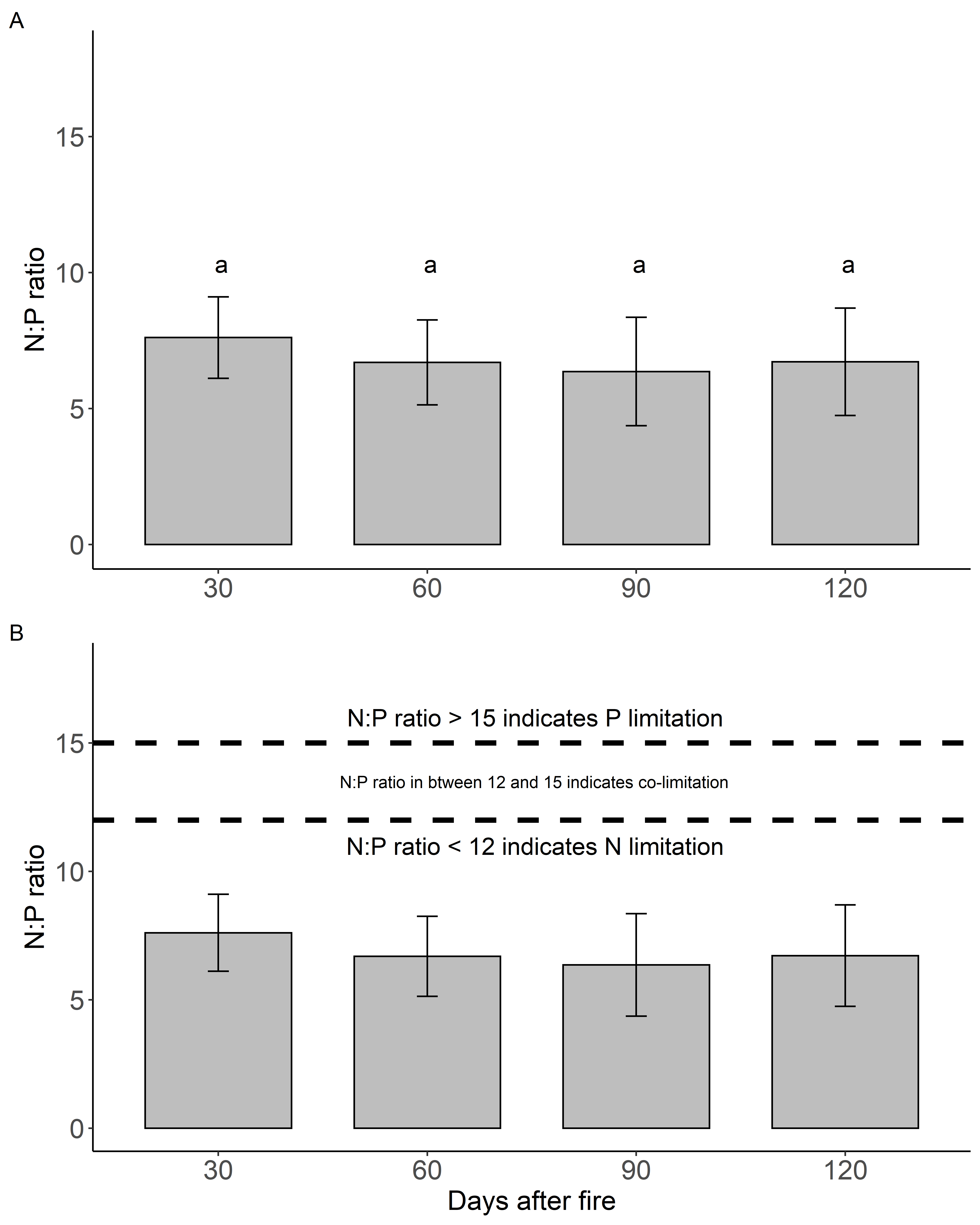

Supplement: Supplementary file 1 — Fig S1 [file ECE3-12-e8794-s005.tiff]

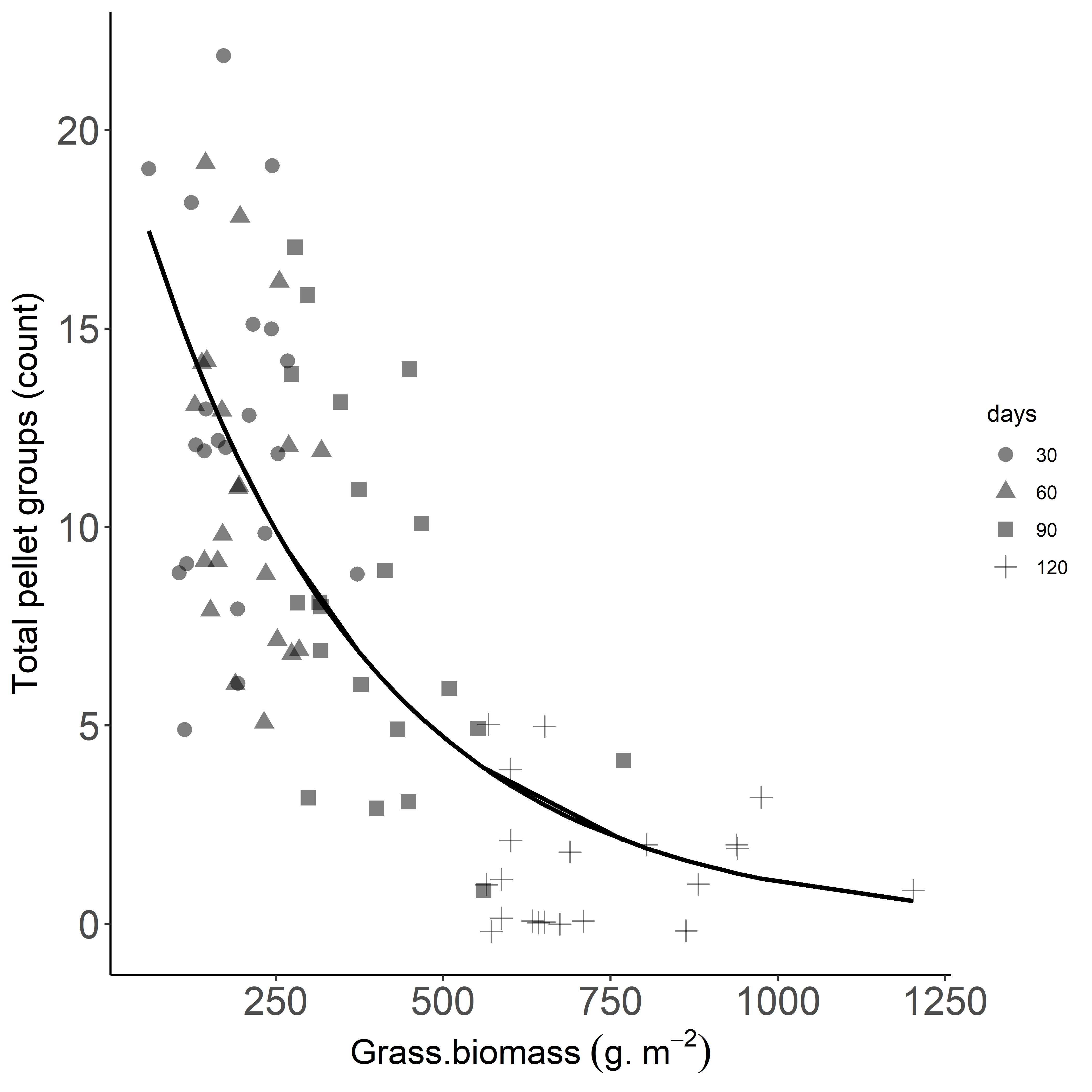

Supplement: Supplementary file 2 — Fig S2 [file ECE3-12-e8794-s006.tiff]

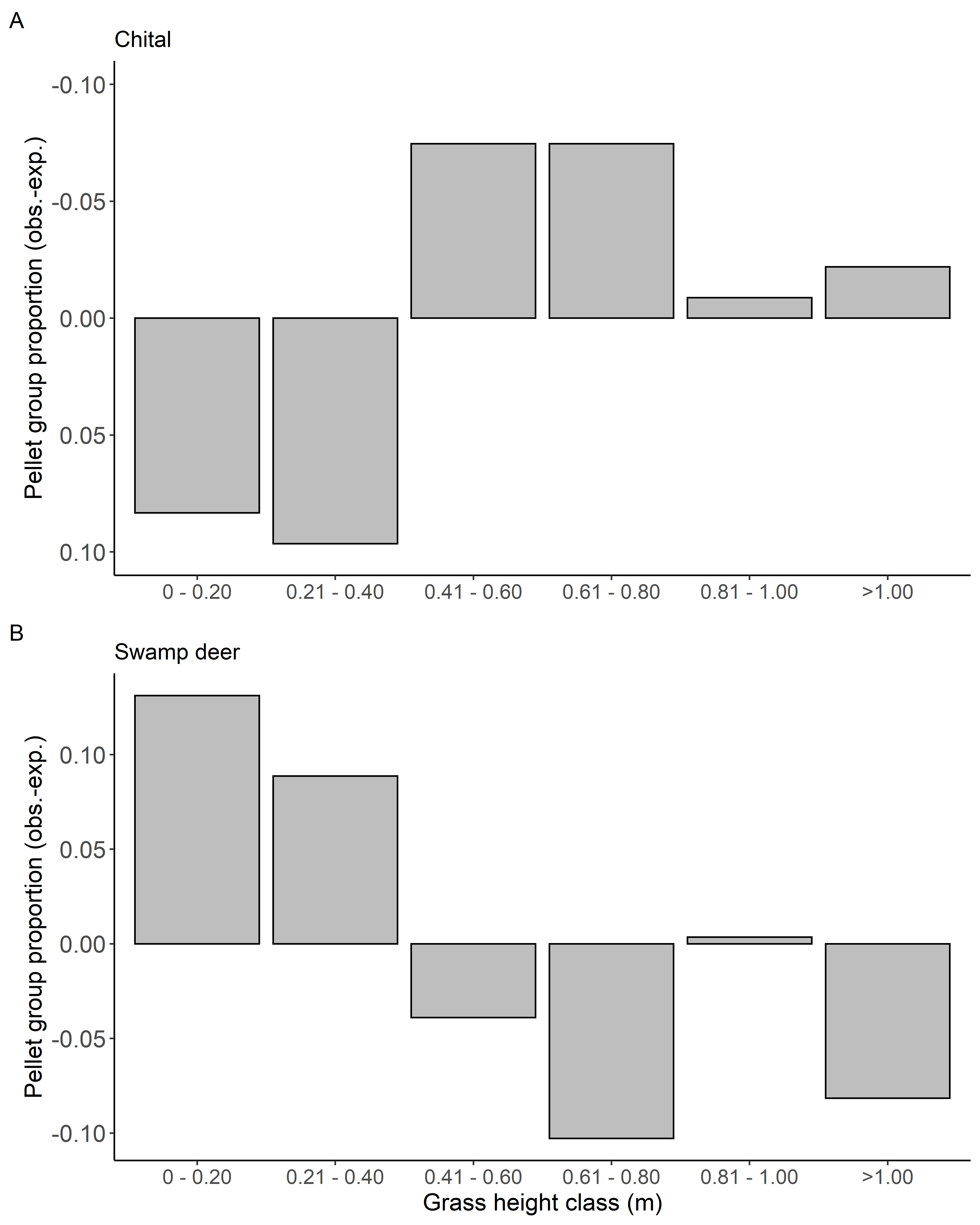

Supplement: Supplementary file 3 — Fig S3 [file ECE3-12-e8794-s004.tiff]

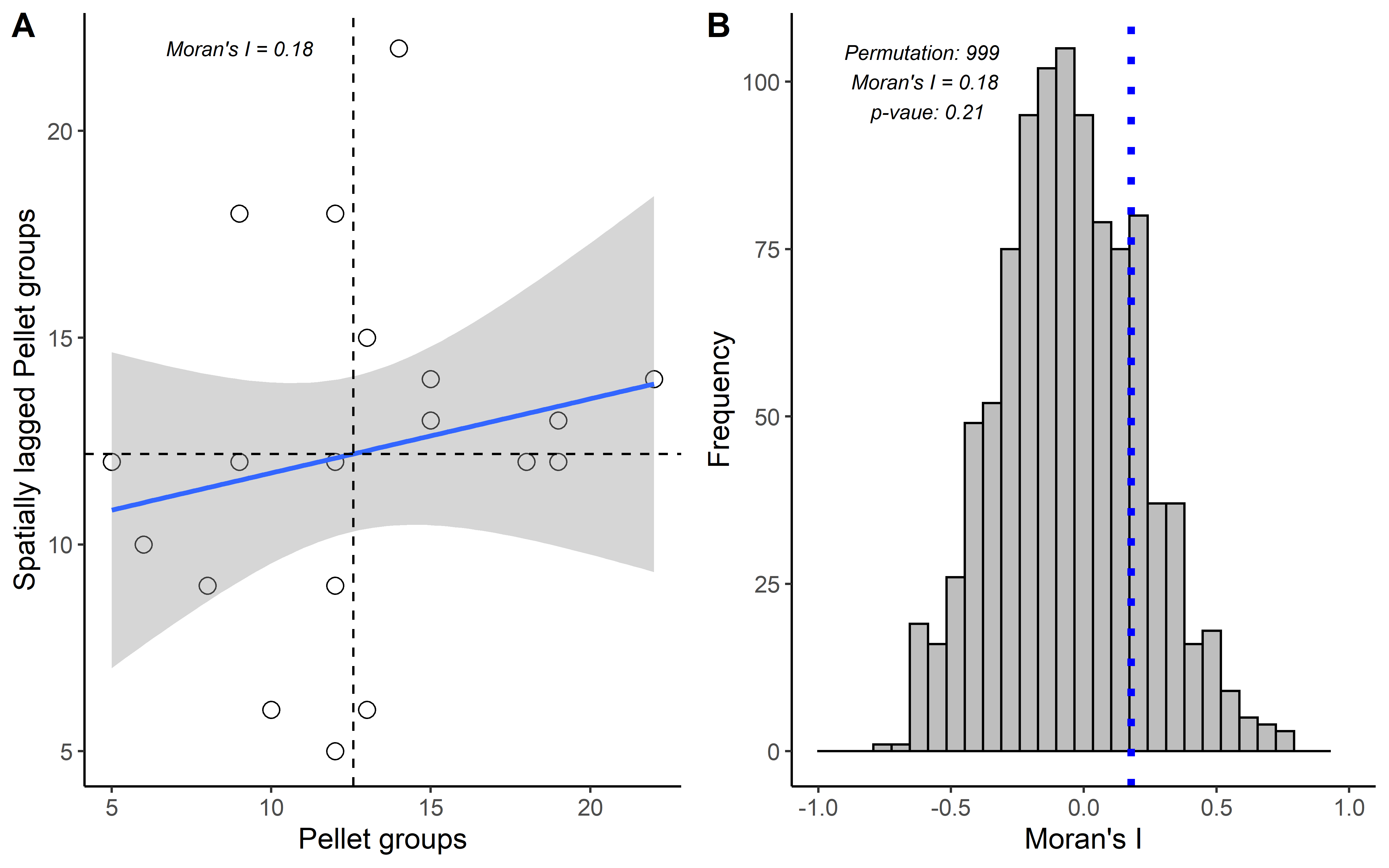

Supplement: Supplementary file 4 — Fig S4 [file ECE3-12-e8794-s002.tiff]

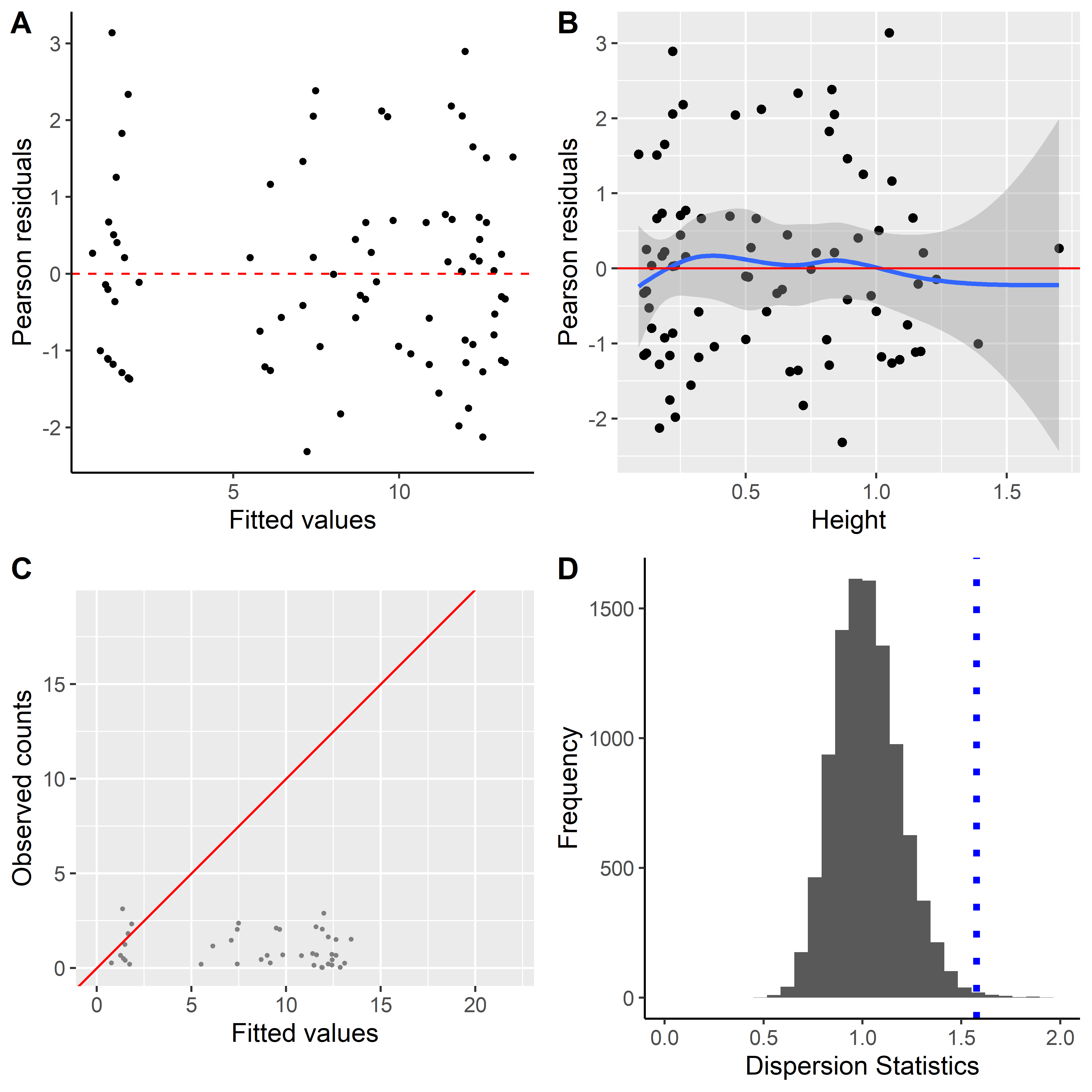

Supplement: Supplementary file 5 — Fig S5 [file ECE3-12-e8794-s001.tiff]

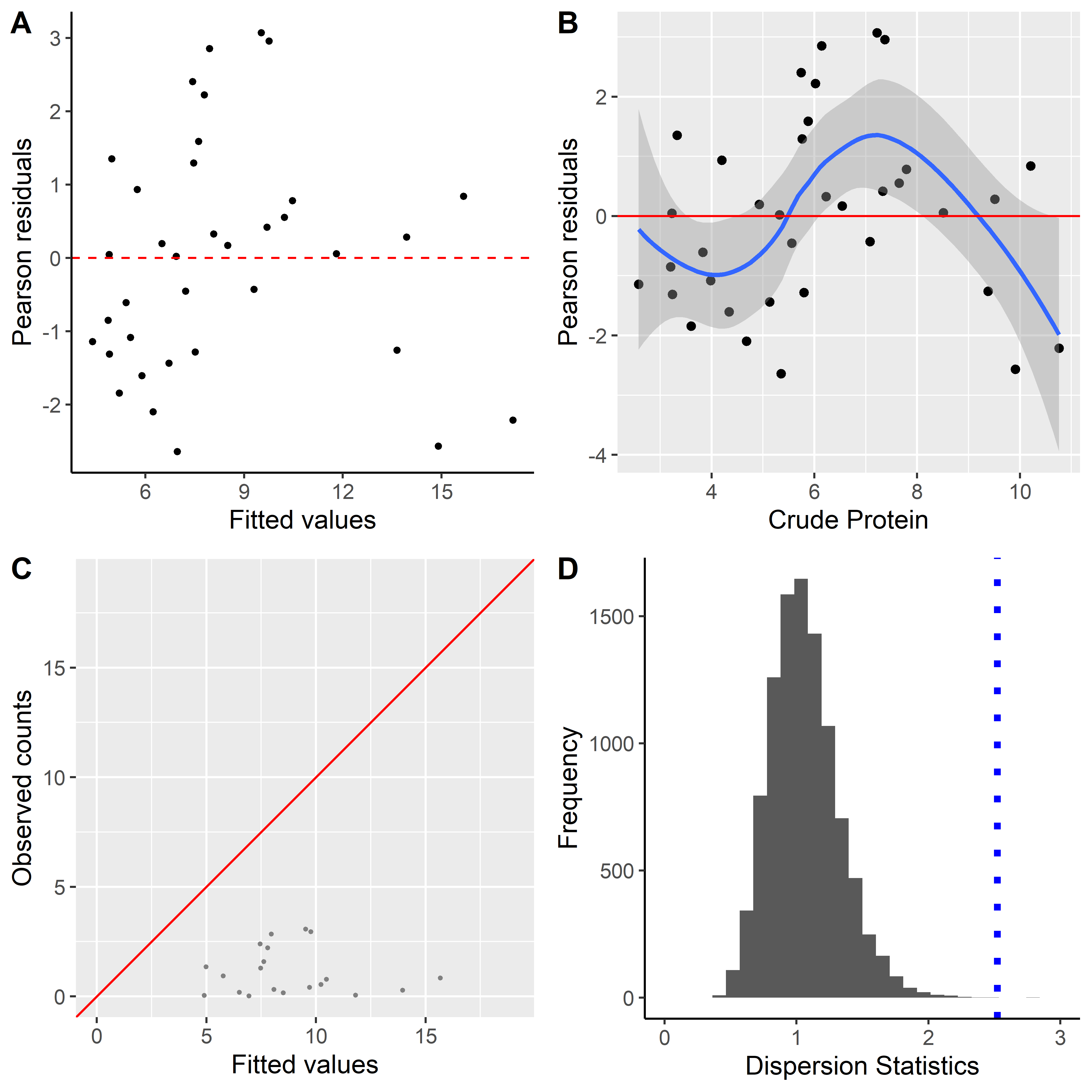

Supplement: Supplementary file 6 — Fig S6 [file ECE3-12-e8794-s003.tiff]

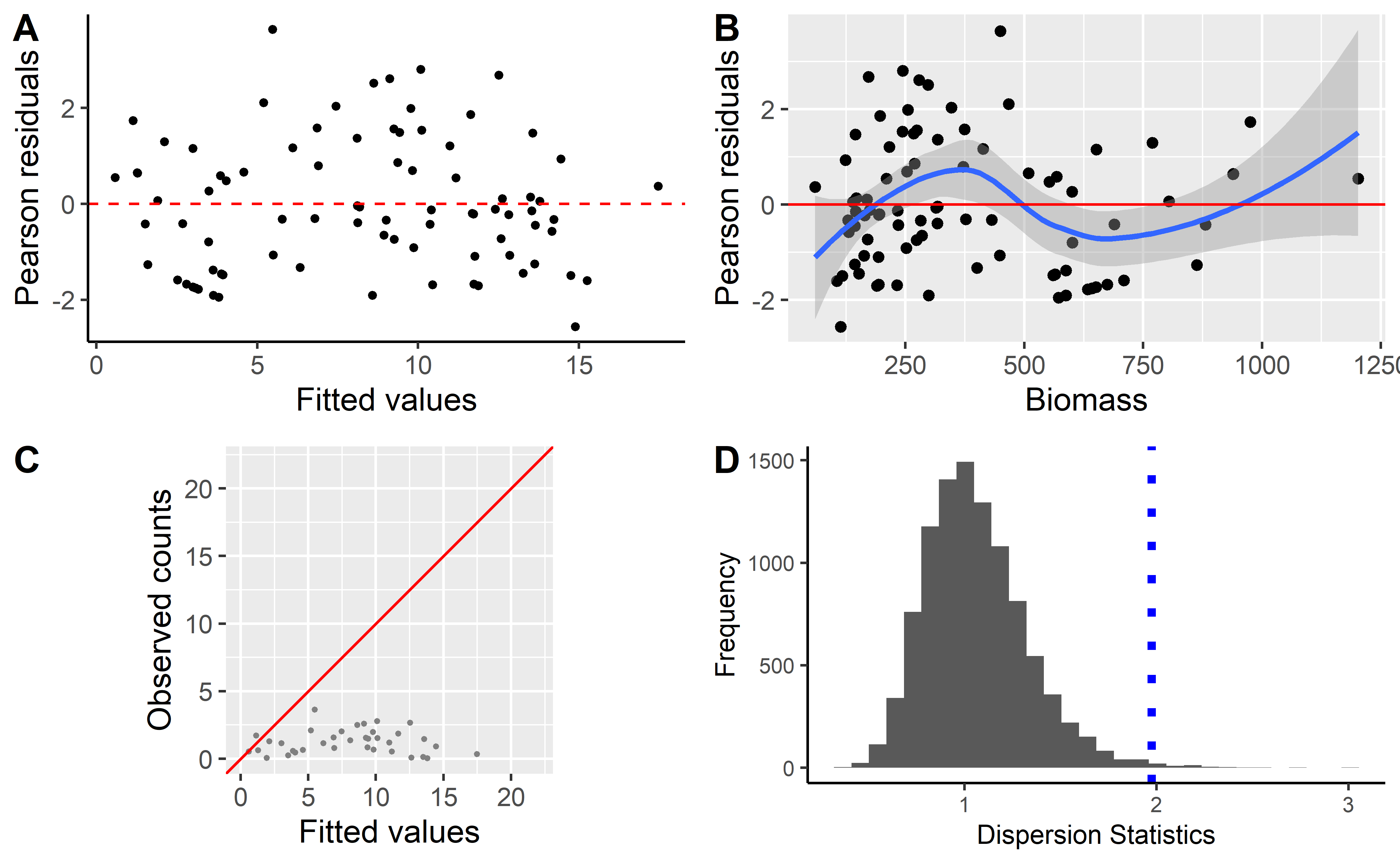

Supplement: Supplementary file 7 — Fig S7 [file ECE3-12-e8794-s008.tiff]

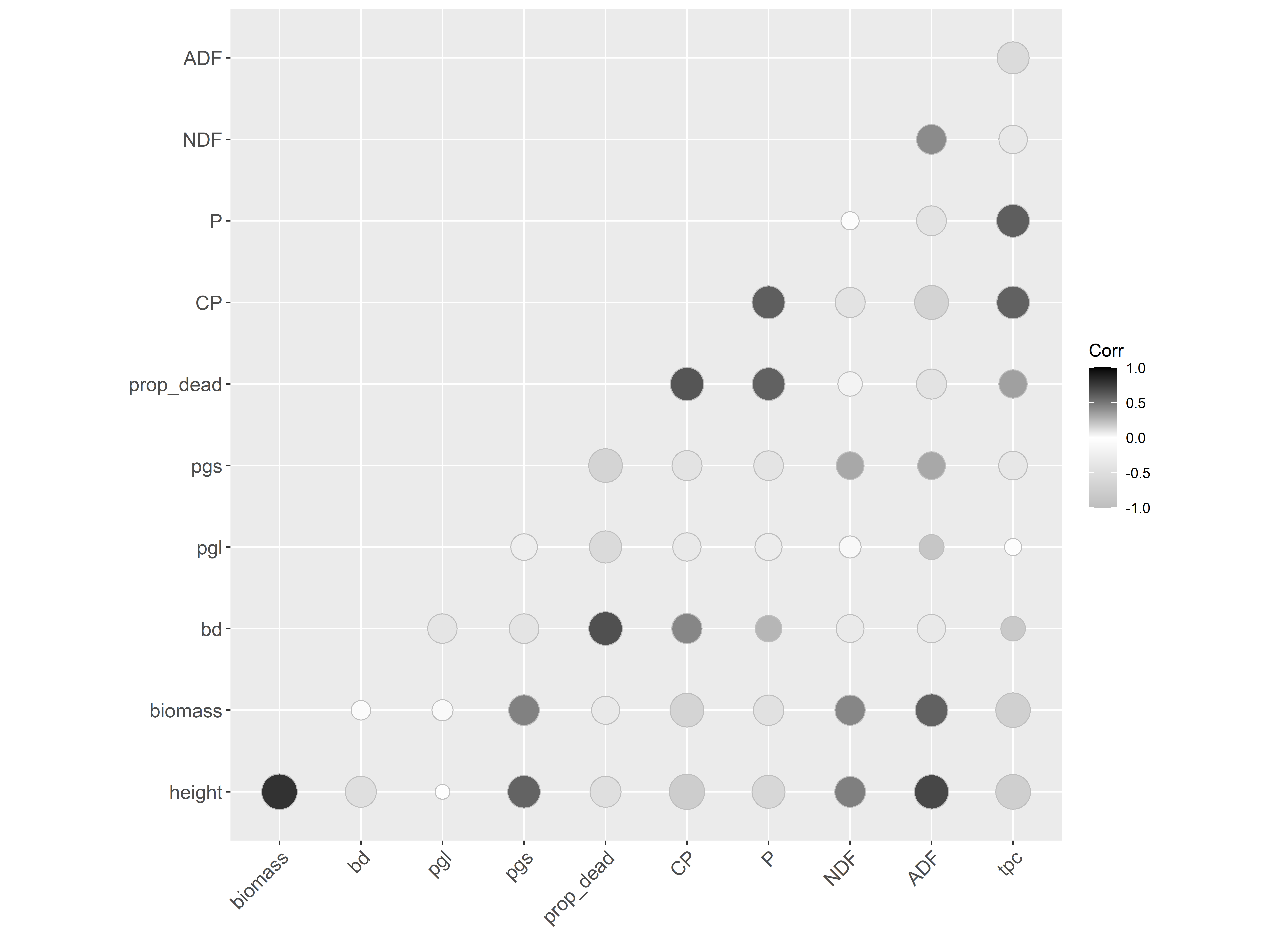

Supplement: Supplementary file 8 — Fig S8 [file ECE3-12-e8794-s007.tiff]
